# A cross-talk between Hepatitis B virus and host mRNAs confers viral adaptation to liver

**DOI:** 10.1038/srep10572

**Published:** 2015-07-17

**Authors:** Jun Hu, Yaxing Xu, Changfei Li, Junli Hao, Shanxin Peng, Xiaoyu Chu, Dake Zhang, Dongping Xu, Songdong Meng

**Affiliations:** 1 CAS Key Laboratory of Pathogenic Microbiology and Immunology, Institute of Microbiology, Chinese Academy of Sciences (CAS), Beijing, China; 2 Laboratory of Disease Genomics and Individualized Medicine, Beijing Institute of Genomics, Chinese Academy of Sciences, Beijing, China; 3 Institute of Infectious Diseases/Liver Failure Medical Center, Beijing 302 Hospital, Beijing, China

## Abstract

Hepatitis B virus (HBV) chronically infects approximately 350 million people worldwide. The replication of HBV which genome is only 3.2 kb long relies heavily on host factors. Previous studies demonstrated that a highly expressed liver-specific microRNA (miRNA) miR-122 suppresses HBV expression and replication in multiple ways. In this study, we found that the miR-122 response elements in viral genome facilitate HBV expression and replication in miR-122 highly-expressed hepatocytes. Moreover, mutations in miR-122 response elements are correlated with viral loads and disease progression in HBV-infected patients. We next found that HBV mRNA with miR-122 response elements alone could lead to altered expression of multiple host genes by whole genome expression analysis. HBV mRNA-mediated miR-122 down-regulation plays a major role in HBV mRNA-induced differential gene expression. HBV mRNA could enhance viral replication via miR-122 degradation and the up-regulation of its target cyclin G1. Our study thereby reveals that under the unique condition of high abundance of miR-122 and viral mRNAs and much lower level of miR-122 target in HBV infection, HBV may have evolved to employ the miRNA-mediated virus and host mRNAs network for optimal fitness within hepatocytes.

Hepatitis B virus (HBV), a major hepatotropic and double-stranded DNA virus, chronically infects 350 million people worldwide. HBV, which genome is only 3.2 kb long, has evolved an extreme adaptation and dependency to its host hepatocytes, and a large number of specific host factors are critical for multiple steps in the HBV replication cycle, including cellular transcription factors, chaperons, and microRNAs (miRNAs)^1^,^2^. The viral genome contains four overlapping open reading frames (ORFs) that efficiently generate pre-C/C or pre-genomic RNA (pgRNA), pre-S, S, and X mRNAs, respectively. These mRNAs encode several viral proteins, including the polymerase, core, HBe, pre-S1, S2, S, and X proteins. Despite its very limited coding capacity, HBV is able to produce enormous numbers of viral mRNAs/proteins and viral loads, as well as a massive surplus of HBs particles, and persist lifelong within infected hepatocytes^3^.

MicroRNAs are a large class of small noncoding RNA molecules of approximately 22 nt in length, which regulate (usually inhibit) target gene expression at the post-transcriptional level. Full-length miRNAs (pri-miRNAs) are transcribed by RNA polymerase II, processed by Drosha, transported into the cytoplasm, and further cleaved by the endonuclease Dicer to produce their mature form miRNAs. Mature miRNA duplexes are loaded onto the RNA-induced silencing complex (RISC), which contains a member of the RNA binding protein Argonaute family (Ago). The guide strand of the miRNA recognizes and binds to complementary target sites in target mRNAs (often in the 3′ untranslated regions (UTRs)) through canonical base-paring between the seed region of approximately 6-8 nt. miRNAs inhibit target gene expression at the post-transcriptional level, either by inducing their deadenylation and degradation or by leading to translational inhibition^4^,^5^. During viral infections, the interaction between miRNAs and their targets viral RNAs plays an essential role in the regulation of viral RNA stability, expression, and translation, as well as viral replication and infection^6^,^7^. Hosts may employ their miRNAs in defense against viral infection by targeting viral mRNAs, whereas viruses also usurp their own miRNAs for the establishment of persistent infection or evasion of immune clearance^8^.

Nevertheless, viral mRNAs harbor common miRNA-binding sequences with host mRNAs^6^,^7^,^8^, suggesting that viral and host mRNAs potentially binds to the same miRNA pool in infected cells. Because miRNA interaction with target mRNAs could lead to miRNA down-regulation, by sequestration/sponge action, destabilization/degradation, or both^9^,^10^,^11^, we hypothesized that viral mRNAs bind and down-regulate specific endogenous miRNAs, and may thereby de-repress and rescue the expression of host target genes within infected cells. Indeed, we and the others show that HBV transcripts directly trigger the down-regulation of miR-122 and miR-15a/16 via the miRNA binding sequences in the viral RNA, and as a consequence, the targets of miR-122 and miR-15a/16 pituitary tumor-transforming gene 1 (PTTG1) binding factor (PBF) and Bcl-2 is up-regulated^12^,^13^,^14^.

As a liver-specific miRNA, miR-122 reaches approximately 70% of the total miRNA population in the adult liver. Although miR-122 has been shown as a host restriction factor to suppress HBV expression and replication in multiple ways^15^, the hepatotropic virus could still efficiently replicate and persist in hepatocytes. In this study, we explore the underlying mechanism on how HBV evolved strategies to establish conditions favoring viral replication and persistence in miR-122 highly expressed hepatocytes. Our results indicate the important virological and clinical founction of HBV mRNAs harboring miR-122 response elements, which enhances viral expression at the expense of adding miR-122 targeting sites.

## Results

### HBV adapts to high miR-122 expression in hepatocytes via miR-122 response elements

**Figure 1:**
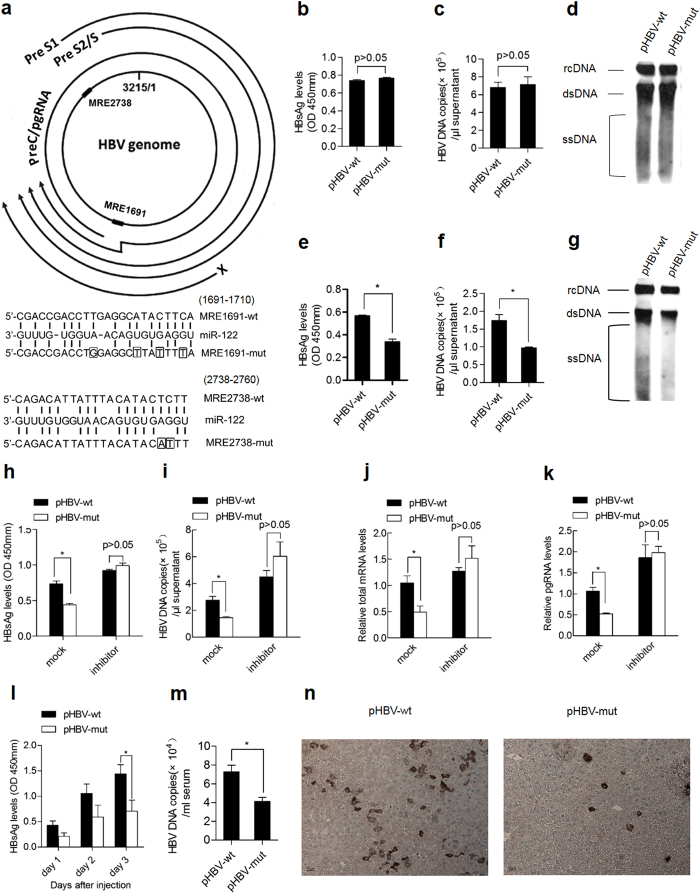
miR-122 response elements facilitate viral replication in hepatocytes with high miR-122 abundance. (**a**) (Top panel) Schematic representation of putative miR-122 response elements in all four HBV mRNAs. ■ represents the miR-122 response elements. (Lower panel) Diagram of predicted miR-122 binding sequence located in the HBV genome. Perfect matches are indicated by a line. Mutations (the frame) were made in the seed region of the miR-122 binding sites. (**b**–**g**) HepG2 (**b**–**d**) and Huh7 (**e**–**g**) cells were transfected with pHBV-wt or pHBV-mut plasmid .The levels of HBsAg in the supernatant were detected by ELISA 48 h after transfection (**b**,**e**) and DNA copies were quantified by real-time PCR (**c,f**) HBV DNA levels in cells were measured by Southern blotting (**d**,**g**) (**h**–**k**) Huh7 cells were co-transfected with pHBV-wt or pHBV-mut plasmid and 122 inhibitor or a randomized oligonucleotide (mock). The secretion of HBsAg was measured by ELISA (**h**) HBV DNA copies (**i**), total mRNA (**j**) and pgRNA levels (**k**) were quantified by real-time PCR. (**l**–**n**) Female BALB/c mice were injected with 15 μg pHBV-wt or pHBV-mut plasmid via the tail vein using the hydrodynamic method (five mice per group). Serum HBsAg was measured at the indicated time point (**l**) HBV DNA copies in serum were determined 3 d after hydrodynamic injection (**m**) HBcAg expression in mice livers was analyzed by immunohistochemical staining 3 d after hydrodynamic injection (**n**) Data shown are the means ± SDs of five mice. Data are representative of two independent experiments. *, P < 0.05; **, P < 0.01.

Previous studies^16^ show that two miR-122 complementary binding sites, spanning nucleotides 1691-1710 and 2738-2760, were found in the 3’-UTR of pre-S1/S2/S mRNAs and coding sequence (CDS) region of pgRNA/core mRNAs, which were designated miRNA response element MRE1691 and MRE2738, respectively (Fig. 1a). To explore the effect of miR-122 response elements on HBV replication, mutations at MRE1691 and MRE2738 as shown in Fig. 1a were introduced in the HBV replication vector pHBV. HepG2 cells which express very low levels of miR-122 were transfected with wild type and mutant pHBV. Similar expression and replication levels were observed between the wild type and mutant viruses (Fig. 1b–d), as mutations were made in the seed region of the miR-122 response elements without changing the amino acid sequence of the pol and X protein. In contrast, when Huh-7 cells which constitutively express miR-122 at high levels^17^ were transfected with the wild type or mutant pHBV, the viral expression (Fig. 1e) and replication (Fig. 1f,g) levels of wide type HBV were much higher than those of the mutant virus. Of note, the promotion effect of miR-122 response elements on HBV expression and replication was completely abolished by depletion of miR-122 by cotransfection of its inhibitor in Huh-7 cells (Fig. 1h–k), indicating that the miR-122 response element-mediated regulation of HBV replication is dependent on miR-122.

We further investigated the effect of miR-122 response elements on HBV replication in vivo. BALB/c mice were hydrodynamically injected with the wild type and mutant HBV replication plasmid. In consistent with the in vitro results, injection of the wild type pHBV resulted in an approximately 2-fold increase in hepatitis B virus s antigen (HBsAg) (Fig. 1l), and viral DNA load (Fig. 1m) in mouse serum compared to that after injection of the mutant pHBV. Immunohistochemical staining results also revealed that the number of HBcAg-positive liver cells in the wild type pHBV-treated mice was greater (~3-fold) than in the mutant plasmid-treated mice (Fig. 1n).

### Mutations in miR-122 response elements correlate with viral loads in HBV-infected patients

**Figure 2:**
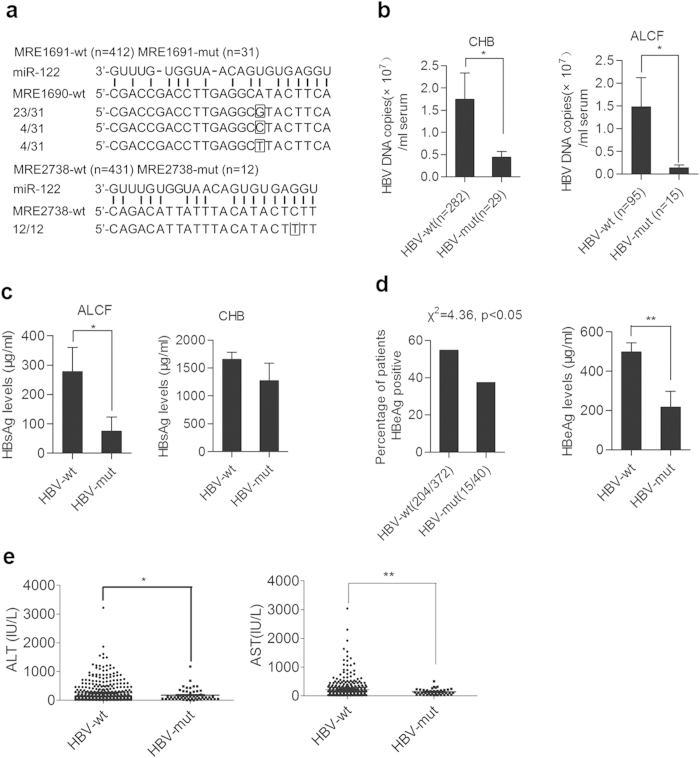
Mutations in viral miR-122 response elements are correlated with viral DNA loads and disease progression in HBV-infected patients. (**a**) The frames indicate mutations in the miR-122 response elements in the HBV genome of virus isolates from HBV-infected patients. The number of patients infected with HBV containing wild type or mutant miR-122 response elements and the mutation frequency in patients are shown. (**b**–**e**) Distribution of serum HBV DNA loads (**b**), HBsAg levels (**c**) HBeAg status (**d**) and ALT and AST levels (**e**) in in CHB and ALCF patients infected with HBV containing wild type or mutant miR-122 response elements. Pearson’s χ^2^ test was used to determine P values. *, P < 0.05; **, P < 0.01.

Clinical and virological assays were performed for 186 mild chronic hepatitis B (CHB-M), 119 severe chronic hepatitis B (CHB-S), and 138 acute-on-chronic liver failure (ACLF) patients. The clinical characteristics of all enrolled patients are described in Table 1. Sequence variations within miR-122 response elements at the seed sequence in the HBV genome in HBV-infected patients are shown in Fig. 2a. The mutation rate in the seed sequence was only 9.7% (43/443) among viruses in patients, which is relatively low. Of note, the miR-122 response elements are highly conserved among different genotypes of HBV by multiple sequence alignment (see Supplementary Table. S1 online), excluding the possibility that the variations in miR-122 response elements are due to different viral genotypes. Compared to patients infected with viral variants containing mutated miR-122 response elements, patients infected with wild type isolates had much higher serum HBV DNA loads (for CHB, 1.746 ± 0.534 × 10^7^ vs. 0.43 ± 0.126 × 10^7^/ml, p = 0.045; for ACLF, 1.481 ± 0.643 × 10^7^ vs. 1.394 ± 0.646 × 10^6^/ml, p = 0.040) (Fig. 2b). Moreover, ALCF patients infected with wild type HBV had higher HBsAg levels than those infected with the variants (277.6 ± 82.68 vs. 75.05 ± 48.56, p = 0.037), and a similar trend was observed for CHB patients, though the difference did not reach statistically significant (Fig. 2c). The hepatitis B virus e antigen (HBeAg)-positive rate in CHB patients with wild type HBV (54.8%) was higher than those with the variants (37.5%). In addition, in HBeAg-positive CHB, patients with wild type HBV had much higher levels of HBeAg than those with variants (p = 0.037) (Fig. 2d), indicating that miR-122 response elements may be correlated with active viral replication. Moreover, the wild type HBV-infected patients had higher alanine aminotransferase (ALT) and aspartate aminotransferase (AST) levels than patients with variants (Fig. 2e). Notably, the miR-122 response element variations did not change the amino acid sequence of HBV in most patients, excluding the possibility that the observed differences were caused by alteration of HBV proteins. There were no significant differences in sex and age between patients with the wild type and variant isolates.

### HBV mRNAs alone lead to aberrant expression of multiple host genes

**Figure 3:**
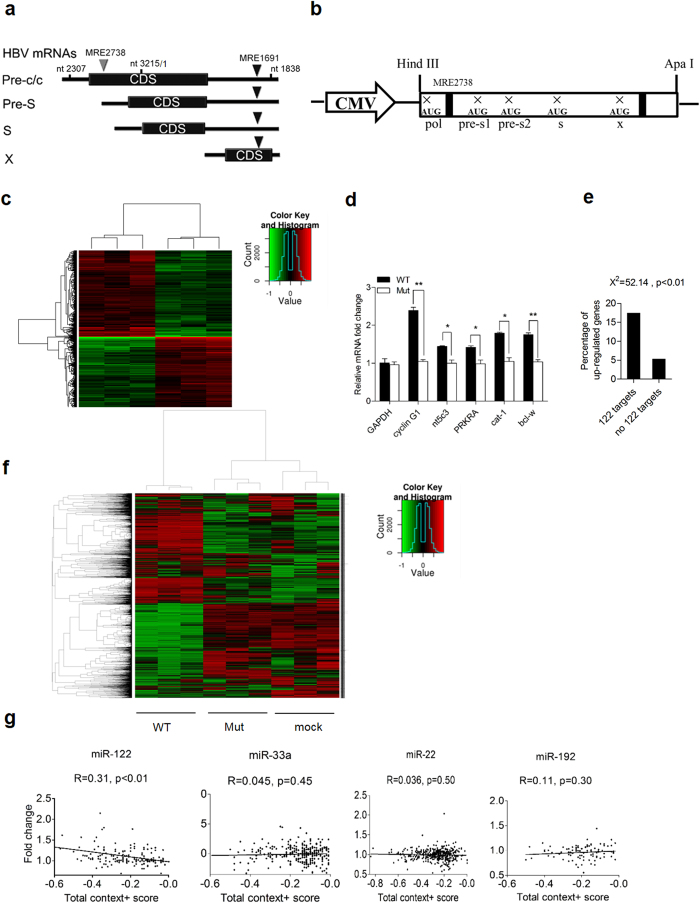
HBV mRNAs affect expression of host genes. (**a**) Schematic representation of the fragment of HBV pgRNA (spanning nucleotides 2307-3215 and 1-1838) used in this study. The fragment contains miR-122 response elements and most sequences of major viral mRNAs. ▼ represents the miR-122 response elements. (**b**) Diagram of HBV pgRNA fragment expression constructs. The fragment of HBV pgRNA (spanning nucleotides 2307-3215 and 1-1838) was inserted into the pcDNA3.1 plasmid, downstream of the CMV promoter, Hind III and Apa I sites. The start codon of Pol, PreS1, PreS2, S and X were mutated, producing the WT construct. (**c**) Shown is hierarchical clustering analysis of transcripts differentially expressed in Huh7 cells transfected with pcDNA3.1 carrying the HBV pgRNA fragment (WT) or the empty pcDNA3.1 vector as a mock. The values from three independent experiments are displayed. (**d**) Quantification of miR-122 targets by real-time PCR in Huh7 cells treated as in c. Data shown are the means ± SD for three independent experiments. *, P < 0.05; **, P < 0.01. (**e**) Distribution of up-regulated transcripts (fold change >1.25) between miR-122 predicted targets and no miR-122 predicted targets. (**f**) Hierarchical clustering analysis of differentially expressed transcripts. Huh7 cells transfected with pcDNA3.1 carrying the wild type (WT) or mutant (Mut) HBV pgRNA fragment within miR-122 response elements or the empty pcDNA3.1 vector as a mock. The values from three independent experiments are displayed. (**g**) The fold change of predicted targets of miR-122, miR-33, miR-22, and miR-192 were plotted based on their context+ scores. Values of the correlation coefficient (R) and P are shown.

To investigate the potential alteration of host gene expression by HBV mRNAs, the HBV pgRNA fragment (spanning nucleotides 2307-3215 and 1-1838) was chosen for analysis as it covers 85% of the whole viral genome and includes most sequences of major viral mRNAs (Fig. 3a). We generated a pcDNA3.1-HBV pgRNA fragment expressing construct (WT) (Fig. 3b). To avoid viral protein expression in this construct, the start codon of Pol, PreS1, PreS2, S and X were mutated as shown in Fig. 3b. Genome-wide expression profiling was performed in Huh7 cells transfected with pcDNA3.1 plasmid containing HBV pgRNA fragment. No HBsAg and HBeAg in the supernatant, and no hepatitis B Core antigen (HBcAg) and viral polymerase in the cells were detected in transfected Huh7 cells (data not shown). Hierarchical clustering of mRNA profiles was performed and visualized with a heat map, where two distinctive expression patterns can be observed (Fig. 3c). These differentially expressed genes may be involved in multiple signaling pathways, including hepatic cholestasis, unfolded protein response, PXR/RXR activation, IL-17 signaling, p53 signaling, TREM-1 signaling, and hepatic fibrosis, etc (see Supplementary Fig. S1 online). These potentially affected signaling pathways are implicated in hepatocyte growth and apoptosis, hepatic lipid and drug metabolism, as well as cytokine production and inflammation. Of note, a large amount of them is related to the pro-inflammatory cytokine TNF signaling cascade (see Supplementary Fig. S2 online). This indicates that HBV mRNAs alone may be involved in regulation of diverse aspects of hepatic function and liver diseases.

Then we searched for putative miRNA targets in the upregualted genes using Targetsan. Intriguingly, the upregulated genes include multiple verified miR-122 targets whose 3’-UTRs contain a common miR-122 response element, such as cat-1, Bcl-w, NT5C3, PRKRA, and cyclin G1^17^,^18^,^19^. Increased expression of these miR-122 targets was verified by real-time PCR analysis (Fig. 3d). The percentage of upregulated genes (fold change >1.25) that are miR-122 targets predicted by Targetscan was much higher than non-miR-122 targets (17.4 vs. 5.3, p < 0.01) (Fig. 3e).

Expression profiling analysis was further conducted in cells transfected with pcDNA3.1 plasmid containing HBV pgRNA fragment with wild type (WT) or mutant (Mut) MRE1691 and MRE2738 as in Fig. 1a. Two distinctive expression patterns can be observed in a heat map (Fig. 3f). Notably, similar gene expression profile was observed in cells transfected with pcDNA3.1 plasmid containing HBV mutant pgRNA fragment and the empty plasmid (mock) (Fig. 3f), indicating that these two miR-122 binding sites play a major role in HBV mRNAs-induced aberrant expression of host genes.

Next, we analyzed the relationship between the de-repression of predicted miR-122 targets and their total context + scores calculated by TargetScan of predicted targets. The predicted targets of miR-122 or other abundant miRNAs in liver (e.g., miR-33a, miR-22, or miR-192) were distributed into context + score bins and plotted against their median fold change. As seen in Fig. 3g, the effect of target de-repression was evident for miR-122 targets but not for targets of any other miRNAs, and the extent of miR-122 target de-repression is correlated with the magnitude of the context + score. These results indicate that the predicted site efficacy between miR-122 and its targets critically contributes to cross-regulation between HBV mRNAs and host mRNAs with shared MREs.

### HBV mRNA enhances viral replication in a protein coding-independent and miR-122 response element-dependent manner

**Figure 4:**
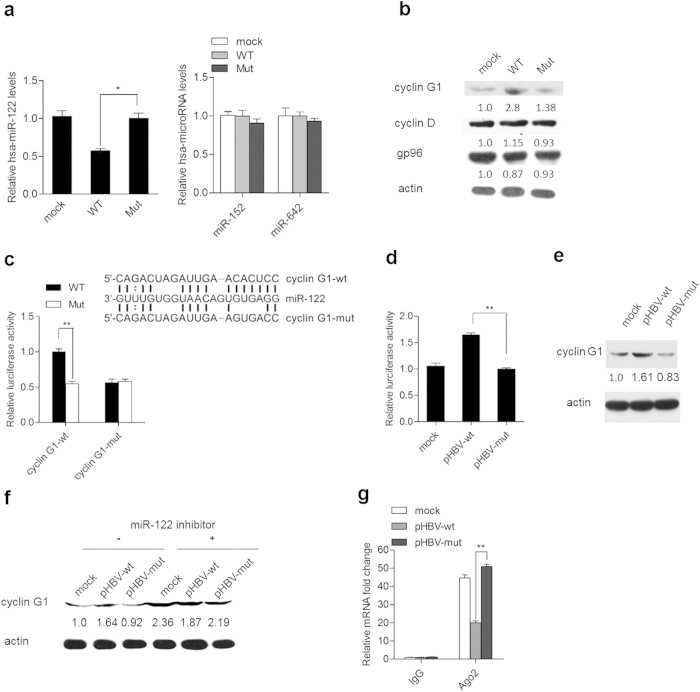
HBV mRNAs up-regulate cyclin G1 in a miR-122 response element-dependent manner. (**a**) Huh-7 cells were transfected with pcDNA3.1 carrying the wild type (WT) or mutant (Mut) HBV pgRNA fragment or with the empty plasmid as a mock, and miR-122 levels were quantified by real-time PCR. The levels of miR-152 and miR-642 were quantified as negative controls. (**b**) Western blot analysis of cyclin G1 in Huh7 cells transfected with pcDNA3.1 carrying the wild type (WT) or mutant (Mut) HBV pgRNA fragment or the empty pcDNA3.1 vector as a mock. The cyclin D and gp96 protein levels were also assessed as negative controls. (**c**) Schematic representation of the miR-122 binding site (cyclin G1-wt) within the 3’UTR of cyclin G1 mRNA; the introduced mutations (cyclin G1-mut) are indicated (the frame). Huh7 cells were co-transfected with pcDNA3.1 carrying the wild type HBV pgRNA fragment and a firefly luciferase reporter plasmid with a 3’UTR containing either a cyclin G1-wt or cyclin G1-mut sequence. At 48 h post-transfection, the firefly luciferase and renilla luciferase activities were measured using a dual-luciferase assay kit. (**d**) Huh7 cells were co-transfected with the cyclin G1 wild type 3’UTR reporter and wild type (pHBV-wt) or mutant (pHBV-mut) HBV replication plasmid or pcDNA3.1 as a mock. At 48 h post-transfection, firefly luciferase and Renilla luciferase activities were measured, and the firefly luciferase activity was normalized to that of Renilla luciferase. (**e**) Huh7 cells were transfected with pHBV-wt, pHBV-mut, or pcDNA3.1 as a mock. At 48 h after transfection, cyclin G1 protein levels were measured by immunoblotting. Actin was used as a loading control. (**f**) Huh7 cells were co-transfected with pHBV-wt, pHBV1.3-mut, or pcDNA3.1 as a mock, along with miR-122 inhibitor or a randomized oligonucleotide (control). Western blot analyses were subsequently performed for cyclin G1. (**g**) Lysates from Huh7 cells transfected with pHBV-wt, pHBV-mut, or pcDNA3.1 as mock underwent immunoprecipitation with anti-Ago2 antibody or control IgG. Immunoprecipitated RNAs were recovered, and quantitative real-time PCR was performed for analysis of miR-122 binding sites in the cyclin G1 3’UTR. Data shown are the means ± SD for three independent experiments. *, *P* < 0.05; **, *P* < 0.01.

Among the cellular genes that are regulated by HBV pgRNA fragment, we focused on cyclin G1 because our previous study shows that cyclin G1 significantly enhances HBV expression and replication by suppressing p53-mediated inhibition of HBV enhancers^20^. As shown in Fig. 4a,b, transfection of HBV pgRNA fragment harboring miR-122 response elements led to a 43% decrease in miR-122 levels in Huh-7 cells, was sufficient to significantly increase cyclin G1 expression levels. HBV pgRNA fragment regulated cyclin G1 in a miR-122 binding-dependent manner, as mutations in miR-122 response elements led to loss of its regulatory function. Meanwhile, no changes were observed for the miR-122 non-targets cyclin D and gp96, indicating that the effect of HBV pgRNA fragment on cyclin G1 was specific. Moreover, HBV pgRNA fragment attenuated endogenous miR-122-mediated inhibition of the luciferase reporter activity containing cyclin G1 3’-UTR (Fig. 4c). As expected, transfection of a wild type but not mutant pHBV in miR-122 response elements de-repressed the inhibition of the cyclin G1 3’-UTR by miR-122 (Fig. 4d) and resulted in an obvious increase in cyclin G1 levels (Fig. 4e). Further, miR-122 depletion by its inhibitor also blocked the ability of pHBV to up-regulate cyclin G1 (Fig. 4f), suggesting that this regulatory effect is miR-122-dependent.

To determine whether HBV mRNAs up-regulate cyclin G1 via direct competition with miR-122, RNA immunoprecipitation (RIP) of Ago2 was performed in Huh7 cells transfected with pHBV. As shown in Fig. 4g, cyclin G1 mRNA was recruited to RISCs at much lower levels in wild type pHBV -transfected cells than in mutant plasmid-transfected cells.

**Figure 5:**
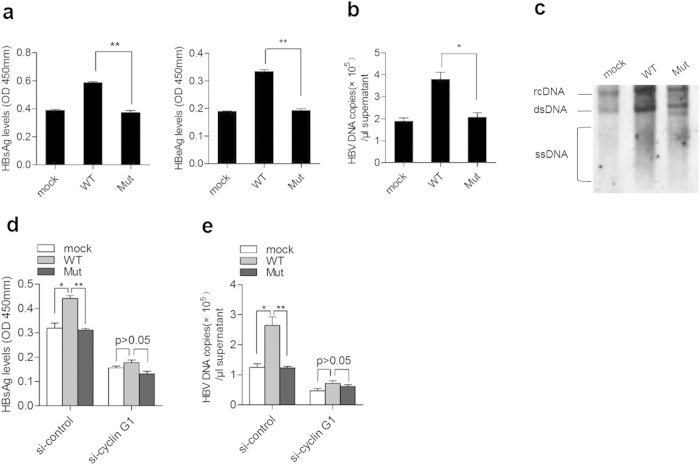
HBV mRNA with miR-122 response elements enhances viral expression and replication through regulation of cyclin G1. (**a–c**) Huh7 cells were co-transfected with HBV replication plasmid (pHBV) and pcDNA3.1 carrying the wild type (WT) or mutant (Mut) HBV pgRNA fragment within miR-122 response elements or the empty pcDNA3.1 vector as a mock. The secretion of HBsAg and HBeAg was measured by ELISA (**a**). HBV DNA copy number in the supernatant was quantified by real-time PCR (**b**) HBV-DNA levels in cells were detected by Southern blotting (**c**) (**d**,**e**) Huh7 cells were co-transfected with HBV replication plasmid (pHBV) and pcDNA3.1 carrying the wild type (WT) or mutant (Mut) HBV pgRNA fragment or the empty pcDNA3.1 vector as a mock, along with cyclin G1 siRNA. HBsAg and HBV DNA levels were determined as in a and b. Data shown are the means ± SD for three independent experiments. * P < 0.05; ** P < 0.01.

As expected, transfection of HBV pgRNA fragment harboring the wild type but not the mutant miR-122 response elements resulted in a significant increase in HBsAg and HBeAg expression compared to the mock (Fig. 5a), as well as an approximately 2-fold increase in HBV-DNA levels (Fig. 5b). A significant enhancement in HBV replication (~4- to 5-fold) was observed in transfected cells by Southern blot analysis (Fig. 5c). In addition, the effect of pgRNA fragment on viral replication was largely abolished under cyclin G1 depletion by siRNA (Fig. 5d,e). These results indicate that HBV mRNAs regulate viral replication in a protein coding-independent and miR-122 response element-dependent manner.

### Dosage of HBV mRNAs and miR-122

**Figure 6:**
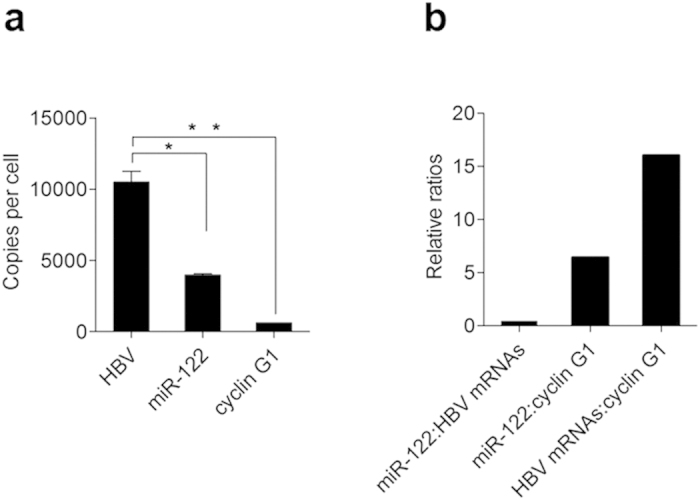
Dosage of HBV mRNAs and miR-122. (**a**) Copies-per-cell analysis of HBV mRNAs, miR-122 and cyclin G1 in Huh 7 cells transfected with the HBV replication plasmid (pHBV). (**b**) The ratios of miR-122 : HBV mRNAs, miR-122 : cyclin G1 mRNA and HBV : cyclin G1 in Huh7 cells transfected with the HBV replication plasmid (pHBV). Data shown are the means ± SD for three independent experiments. * P < 0.05, ** P < 0.01.

An important consideration in the HBV mRNAs-miR-122 interaction is the absolute level of HBV mRNAs and miR-122 transcripts in cells^21^, so we measured the copy numbers of these factors per Huh7 cell. Quantitative real-time PCR measurements calibrated with an internal standard curve of synthetic miR-122 revealed that miR-122 was expressed at ~4000 molecules per Huh7 cell (Fig. 6a), which is comparable to levels previously reported^22^. Meanwhile, we also measured the HBV mRNA abundance after transfecting Huh7 cells with pHBV. The transfection introduced up to 10,000 HBV mRNA transcripts per cell, which is comparable to that of CHB patients^12^,^23^. This suggests that HBV mRNAs can functionally engage miR-122. In contrast, cyclin G1 mRNA was expressed at only ~625 molecules per Huh7 cell by real-time PCR analysis. A Low ratio of miR-122 : HBV mRNAs and high ratios of miR-122 : cyclin G1 mRNA and HBV : cyclin G1 mRNAs were displayed in pHBV-transfected Huh-7 cells (Fig. 6b), which indicates interaction between HBV mRNAs and cyclin G1 mRNA may occur under such a circumstance.

## Discussion

We and others previously reported that HBV mRNAs harboring specific miRNA binding sites may bind and down-regulate these miRNAs, leading to up-regulation of the miRNA targets which are involved in cell apoptosis and hepatocellular carcinoma (HCC) development. The preliminary data suggest a possible interaction between HBV mRNAs and host mRNAs. In this study, we show that HBV may have evolved to adapt to the high expression levels of miR-122 in the liver environment via viral miR-122 response elements. HBV mRNA alone enhances viral expression and replication and contributes to viral persistence. Next, we profiled the potential host genes which expression could be epigenetically regulated by HBV mRNA using whole genome expression analysis. Our data demonstrate a potential viral mRNA-mediated signaling network in hepatocytes that is involved in cell growth and apoptosis, hepatic lipid metabolism, and pro-inflammation. Further analysis shows that HBV mRNA-mediated miR-122 down-regulation plays an important role in HBV mRNAs-induced altered expression of host genes. Finally, we assessed the reciprocal interactions among HBV, miR-122 and cyclin G1 with quantitative measurement of abundance of miR-122, HBV and the miR-122 target cycline G1 mRNAs.

The basis for miRNA mediated-virus and host mRNAs crosstalk is determined by base pairing complementarity, miRNA binding/recognition, the ability of viral mRNAs to sequester and degrade miRNAs, and the extent of de-repression in host mRNAs by miRNA inhibition. Therefore, the relative abundance of virus vs. host mRNAs, levels of miRNAs, and the number of miRNA response elements may all contribute to virus-host interactions, according to a mathematical mass-action model for competitive endogenous RNA (ceRNA) networks^24^. Long noncoding RNAs (lncRNAs) and pseudogenes can function as a decoy for microRNAs that target protein-coding genes due to their ability to compete for miRNA binding and to de-repress expression of these genes^25^,^26^,^27^. The crosstalk between ceRNAs (e.g., protein-coding and non-coding mRNAs) through competition for their shared miRNAs is involved in signaling pathways and networks in human diseases, such as autoimmune diseases and cancer. However, Denzler *et al.*^21^ pointed out that in a primary hepatocyte infection model miR-122-mediated ceRNA occurs in a threshold-like manner at high target site abundance (≥1.5 × 10^5^ added target sites per cell). In the present study the transfection of HBV replication plasmid introduced up to 10,000 HBV mRNA transcripts per Huh7 cell (Fig. 6a), which is comparable to the viral mRNA copy number per cell in active HBV replication of clinical viral infection^20^,^23^. The number of HBV transcripts in infected hepatocytes far exceeds the number of most host mRNAs. Meanwhile, miR-122 is one of the most abundant miRNAs discovered so far, accounting for about 70% of the whole hepatic miRNome in human^15^. Moreover, unlike ceRNA networks in which miRNAs levels are usually unchanged under sequestration by endogenous mRNAs^28^, miR-122 level is significantly decreased by overexpression of HBV mRNAs (Fig. 4a). Reduced miR-122 levels were also observed during HBV infection, as well as in HBV-infected HCC^13^,^17^,^20^. In addition, our results demonstrate that the extent of up-regulation of miR-122 targets by HBV mRNA is correlated with their miRNA predicted site efficacy (Fig. 3f). In agreement with our results, recent studies provide compelling evidence that virus-expressed coding and non-coding RNAs are involved in the degradation and decay of host miRNAs^10^,^12^,^29^. A recent study showed that intergenic RNA sequences harboring a miR-17-92 binding site in the genome of virulence of human cytomegalovirus (HCMV) led to selective, sequence-specific turnover of mature miR-17 and miR-20a, but did not repress viral intergenic RNA expression^30^. Taken together, we hypothesize that in HBV infection, excessive viral mRNAs, all of which harbor one or even two miR-122 response elements, effectively sequestrate miR-122 and lead to its destabilization and degradation. In this specific pathogenic settings, conditions might arise in which HBV mRNAs-induced miR-122 alteration may cause significant effect on target gene (e.g. cyclin G1) expression. This deserves detailed studies.

As a highly prevalent hepadnavirus, HBV can evolve by mutations to increase its adaptation against environmental selection (e.g., immune-evasion or drug resistance)^31^,^32^,^33^,^34^. From our current preliminary data, we speculate that positive reciprocal regulation and cross-talk between HBV mRNAs and host mRNAs via the common miRNA response elements forms a complex virus-host network. HBV usually generates highly redundant transcripts upon infection. Conceivably, to establish infection, HBV likely evolved to increase the number of shared miRNA response elements with supportive host factors that facilitate virus infection or replication and decrease the number shared with inhibitory host that factors that restrict viral infection. Further comprehensive studies, such as RNA-seq analysis of crosslinked RNAs, are needed to explore the miRNA-mediated interaction between virus and host mRNAs.

Our study suggests that under infection, HBV mRNAs may disturb host mRNA homeostasis through miR-122-mediated networks. Viruses may employ either strategy (to usurp interaction with miRNAs or to avoid miRNA targeting) to establish persistent infection, depending on which strategy plays a major role in favoring viral infectivity and replication. Information regarding the balance between miRNA pools and host mRNA homeostasis under HBV infection is essential to provide further dissection of the impact of the miRNA-mediated networks on virus and host interactions. Further studies on the cross-regulation between HBV mRNAs and host mRNAs may help to elucidate the molecular basis for viral persistence and pathogenesis, which may facilitate the development of new antiviral strategies.

## Methods

### Ethics Statement

For human subjects: written informed consent was provided by all study participants.

The study protocol was approved by the ethics committee of Beijing 302 Hospital.

The mouse experimental design and protocols used in this study were approved by the Research Ethics Committee of Institute of Microbiology, Chinese Academy of Sciences (permit number PZIMCAS2011001). Animals received humane care, and the study of mice was in strict accordance with “the regulation of the Institute of Microbiology, Chinese Academy of Sciences of Research Ethics Committee.”

### Patients

A total of 443 patients from Beijing 302 Hospital were included in the study. The variations of the two miR-122 response elements in the HBV genome were analyzed with clinical information in the database of records for HBV-infected patients built by the Beijing 302 Hospital (Beijing, China) (http://www.hbvdb.com). The diagnosis criteria for CHB and ACLF complied with the 2006 Diagnostic and Treatment Guidelines for Liver Failure issued by the Chinese Society of Infectious Diseases and Chinese Society of Hepatology, Chinese Medical Association^20^. Briefly, CHB-M patients defined as those who had chronic HBV infection with HBsAg positivity for 6 months and a histopathological diagnosis of mild liver injury with compatible laboratories or ultrasonographics. CHB-S patients met the following criteria: symptoms of severe liver injury; significant elevation of ALT and significant alteration of biochemical parameters that includes at least one of the following: serum albumin level of 32 g/liter, serum total bilirubin (TBIL) level of 85.5 mol/liter, plasma prothrombin activity (PTA) of 40 to 60%, and serum cholinesterase level of 4,500 IU/liter. The diagnostic standard for ACLF mainly includes a history of CHB with acute hepatic insult manifesting as jaundice and severe digestive problems with the following criteria: 10 times the normal level of TBIL level (>171 mol/liter) and decreasing PTA (<40%).

### Plasmid constructs

The HBV replication plasmid pHBV1.3, containing 1.3 copies of the HBV genome (genotype C), was maintained in the lab. Mutations in the miR-122 response elements were introduced into pHBV1.3, and the resulting plasmid was designated as pHBV-mut. HBV pgRNA fragments (spanning nucleotides 2307-3215 and 1-1838) containing the miR-122 response elements were PCR amplified from pHBV 1.3 and cloned into the pcDNA 3.1 vector. The start codon of Pol, PreS1, PreS2, S and X in the HBV pgRNA fragments were mutated. The resulting plasmid was designated as WT. miR-122 response elements in WT were mutated by site-directed mutagenesis following the manufacturer’s instructions (Stratagene) and designated as Mut. The 3’-UTR of cyclin G1 containing the miR-122 binding site was amplified by PCR using the following primers: forward, 5’-GCTCTAGAGCCTCAAACTGAATCCCATCAAG-3’; reverse, 5’- GCTCTAGACCGCTCGAGTTTTGGCACAGTAAGGGCATC-3’. The PCR product was cloned into the 3’UTR of the firefly luciferase gene in the pGL3 plasmid.

### Mice

Female BALB/c mice were purchased from Vital River Laboratories, Beijing. A total of 15 μg wild type (pHBV-wt) or mutant (pHBV-mut) HBV replication plasmid was injected into the mouse tail vein via hydrodynamic injection. Three days after injection, mice were sacrificed. Sera were assessed for HBsAg and HBV DNA levels. HBcAg in liver samples was detected with an immunohistochemical staining assay.

### Reagents

The chemically synthesized cyclin G1-specific siRNA, miR-122 mimic, and non-specific control, as well as the miR-122 inhibitor and non-specific control, were purchased from RiboBio Co., Ltd. (Guangzhou, China). The sequences of the cyclin G1 siRNA was: sense, 5’-TGTCCCATTGGCAACTGAC dTdT; antisense, 5’-GUCAGUUGCCAAUGGGACA TdTd. Rabbit anti-human cyclin G1 (clone F-5) was from Santa Cruz Biotechnology.

### Cell culture and transfection

Human hepatoma cell lines HepG2 and the 293T cell line were obtained from the ATCC (Manassas, VA, USA). Huh7 cells were maintained in the lab. Transfections were performed using Lipofectamine 2000 reagent (Invitrogen).

### Real-time PCR

Total RNA was extracted with Trizol Reagent, and quantified by real-time PCR using the SYBR Green Premix Reagent (Takara Bio Inc., Shiga, Japan) with a GAPDH internal control for normalization. The following forward and reverse primers were used: human GAPDH forward, 5’-AGAAGGTGGTGAAGCAGGCGTCG-3’ and reverse, 5’-CCTTGGAGGCCATGTGGGCC-3’; cyclin G1 forward, 5’-AATGAAGGTACAGCCCAAGCA-3’ and reverse, 5’-GCTTTGACTTTCCAACACACC-3’; CAT-1 forward, 5’-CTTCATCACCGGCTGGAACT-3’ and reverse, 5’-GGGTCTGCCTATCAGCTCGT-3’; Bcl-w forward, 5’-GAGCCATATAGTTCCTTGGGA-3’ and reverse, 5’-TAGAATAAGTGGGGAGTGGGA-3’; PRKRA forward, 5’-ACGAATACGGCATGAAGACC-3’, and reverse, 5’-TGGAAGGGTCAGGCATTAAG-3’; NT5C3 forward, 5’-AAGAATGGCAGATGGAGTGG-3’ and reverse, 5’-ACAGTTCAATTGCACCCACA-3’; HBV pgRNA forward, 5’-TCTTGCCTTACTTTTGGAAG-3’ and reverse, 5’-AGTTCTTCTTCTAGGGGACC-3’; and total HBV RNA forward, 5’-CTCCCCGTCTGTGCCTTCTC-3’ and reverse, 5’-TCGGTCGTTGACATTGCTGA-3’.

### TaqMan miRNA analysis

Real-time PCR analysis for miR-122 was performed using a TaqMan miRNA Kit (Applied Biosystems). The U6 endogenous control was used for normalization. Pri-miR-122 was detected using a High Capacity cDNA Reverse Transcription Kit (Applied Biosystems) and a TaqMan pri-miRNA Assay. Actin was used as an internal standard gene. Relative expression was quantified using the comparative threshold cycle (Ct) method.

### Luciferase assay

Huh-7 cells were transfected with a firefly luciferase reporter plasmid and pRL-TK as the control. Firefly luciferase and Renilla luciferase activities were measured, and the firefly luciferase activity was normalized to that of Renilla luciferase after 48 h.

### Western blotting

Western blot analyses were performed as described^20^.

### Southern blotting

Southern blots for core particle-associated HBV DNA were performed as described^17^.

### Detection of HBsAg and HBeAg

The expression levels of HBsAg and HBeAg were measured by ELISA as described previously^17^.

### Transcriptional profiling by microarray

Comparative microarray analysis of mRNA for Huh7 cells transfected with pcDNA3.1, WT, or Mut was performed on GeneChip®PrimeView™ Human Gene Expression arrays (Affymetrix) following the manufacturer’s protocol by Shanghai Biotechnology Corporation. Corresponding microarray expression data were analyzed by pairwise differences determined with the Student-t test (p < 0.05). Further analyses of the differentially expressed genes, including network, pathway, and functional analysis, were performed by the Shanghai Biotechnology Corporation.

### RNA immunoprecipitation

A total of 5 × 10^6^ Huh7 cells was transfected with pcDNA3.1, WT, or Mut. After 24 h, cells were washed twice with 10 ml of ice cold phosphate-buffered saline (PBS). The cells were resuspended in 5 ml lysis buffer (25 mM Tris HCl (pH 7.5), 150 mM KCl, 2 mM EDTA, 0.5% NP-40, and 0.5 mM DTT) with RNase inhibitors and protease inhibitors. After a 30-min incubation, lysates were pelleted by centrifugation at 20,000 g for 30 min at 4 °C. Ago2-antibody (6μg; abcam, ab32381) or control antibodies (immunoglobulin G (IgG)) were added to 2.5 mL RPMI-medium and incubated with 30 μl Protein-G Sepharose beads (GE Healthcare) for 12–18 h with tumbling end over end at 4 °C. The beads were pelleted and wash four to five times with cold lysis buffer. The beads were subsequently incubated with 5 mL cell lysate for 2.5 h under constant rotation at 4 °C. After incubation, the beads were washed four times with IP wash buffer containing 300 mM NaCl, 50 mM Tris HCl (pH 7.5), 5 mM MgCl2, 0.1% NP-40, and 1 mM NaF and once with PBS to remove residual detergents. The AGO2 components were released and RNA was isolated from the immunoprecipitated pellet by adding Trizol reagent. Total RNA was used for qRT–PCR of cyclin G1 mRNAs.

### Transcript copy-number analysis

RNA was extracted from 1 × 10^5^ Huh7 cells using Trizol Reagent. Absolute quantification of HBV total mRNAs and mir-122 was performed by qRT–PCR. For a standardized evaluation, threshold cycle (CT) values were compared to a 10-fold serial dilution of either in vitro-transcribed HBV mRNAs or synthetic miR-122 mimics (RiboBio Co., Ltd). Then, the copies of transcript per cell were calculated with standard stoichiometric methods.

### Statistical analyses

Data were expressed as mean ± SD (standard deviation) from three independent experiments. The statistical significance between two and more than two groups was measured using the two-tailed Student’s t-test and ANOVA respectively. Association between variables was assessed by Pearson’s χ^2^ test. P values < 0.05 were considered significant.

## Additional Information

**How to cite this article**: Hu, J. *et al.* A cross-talk between Hepatitis B virus and host mRNAs confers viral adaptation to liver. *Sci. Rep.* **5**, 10572; doi: 10.1038/srep10572 (2015).

**Accession number**: The Gene Expression Omnibus accession number for the microarray data reported in this paper is GSE64084.

## Supplementary Material

Supplementary Information

## Figures and Tables

**Table 1:** Clinical and virologic characteristics of CHB-M, CHB-S, and ACLF patients.

**characteristic**	**CHB-M**	**CHB-S**	**ALCF**	**P value**
No. of patients	186	119	138	
Mean ± SD age (yr)	40.82 ± 13.46	38.65 ± 13.23	46.28 ± 11.87	<0.01
Gender (no. of M/no. of F)	162/24	101/18	102/36	0.0575
No.(%) of patients with the miR-122 MRE variation	16(8.6)	12(10.1)	15(10.9)	0.976
No.(%) of patients with HBeAg positive	115(61.8)	49(41.2)	39(28.3)	<0.01
No. of HBV DNA copies/ ml				
Mean	1.64 × 10^7^	1.39 × 10^7^	1.3 × 10^7^	0.682
Range	1020-1.6 × 10^9^	500-2.64 × 10^8^	505-5.3 × 10^8^	
Mean ± SD (IU/liter)				
ALT	128.8 ± 192.1	456.5 ± 377.1	257.7 ± 388.4	<0.01
AST	99.2 ± 114.3	289.3 ± 269.6	293. ± 426.4	<0.01

HBV, hepatitis B virus; CHB-M, mild chronic hepatitis B; CHB-S, severe chronic hepatitis B; ACLF, acute-on-chronic liver failure; M, male; F, female; HBeAg, hepatitis B virus e antigen; ALT, alanine aminotransferase; AST, aspartate aminotransferase.
