# Supplementary material for: A cross-talk between Hepatitis B virus and host mRNAs confers viral adaptation to liver
Source: Sci Rep. 2015 Jul 17;5:10572. doi: 10.1038/srep10572 (PMC4505342; doi:10.1038/srep10572)
Supplement: Supplementary Information [file srep10572-s1.pdf]

A cross-talk between Hepatitis B virus and host mRNAs confers viral adaptation to liver

Jun Hu[1], Yaxing Xu[1], Changfei Li[1], Junli Hao[1], Shanxin Peng[1], Xiaoyu Chu[1], Dake Zhang[2], Dongping Xu[3], Songdong Meng[1,*]

[1] CAS Key Laboratory of Pathogenic Microbiology and Immunology, Institute of Microbiology, Chinese Academy of Sciences (CAS), Beijing, China

[2] Laboratory of Disease Genomics and Individualized Medicine, Beijing Institute of Genomics, Chinese Academy of Sciences, Beijing, China

[3] Institute of Infectious Diseases/Liver Failure Medical Center, Beijing 302 Hospital, Beijing, China

*corresponding: mengsd@im.ac.cn

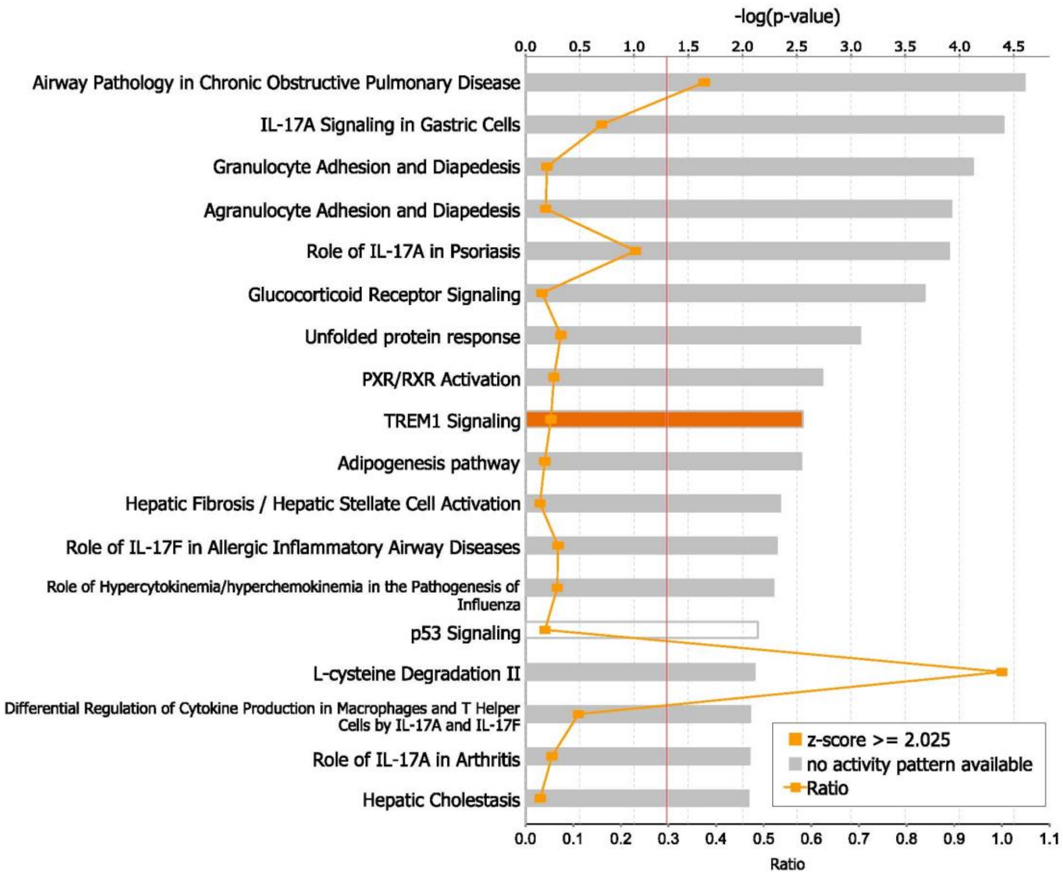

Supplementary Fig. S1 Top ingenuity canonical pathways enriched in differentially expressed genes identified by microarray.

Huh7 cells transfected with pcDNA3.1 carrying the wild type (WT) or mutant (Mut) HBV pgRNA fragment within miR-122 response elements. Differentially expressed genes were identified by microarray. Colored bars indicate the significance of differentially expressed gene enrichment in the canonical pathways (-log of p-value, Fisher's exact test right-tailed) and their predicted activation status according to the z-score (orange, activation; grey, no prediction can currently be made; white, z-score at or very close to 0). Meanwhile, the orange lines represent the ratio of differentially expressed genes in the given pathway.

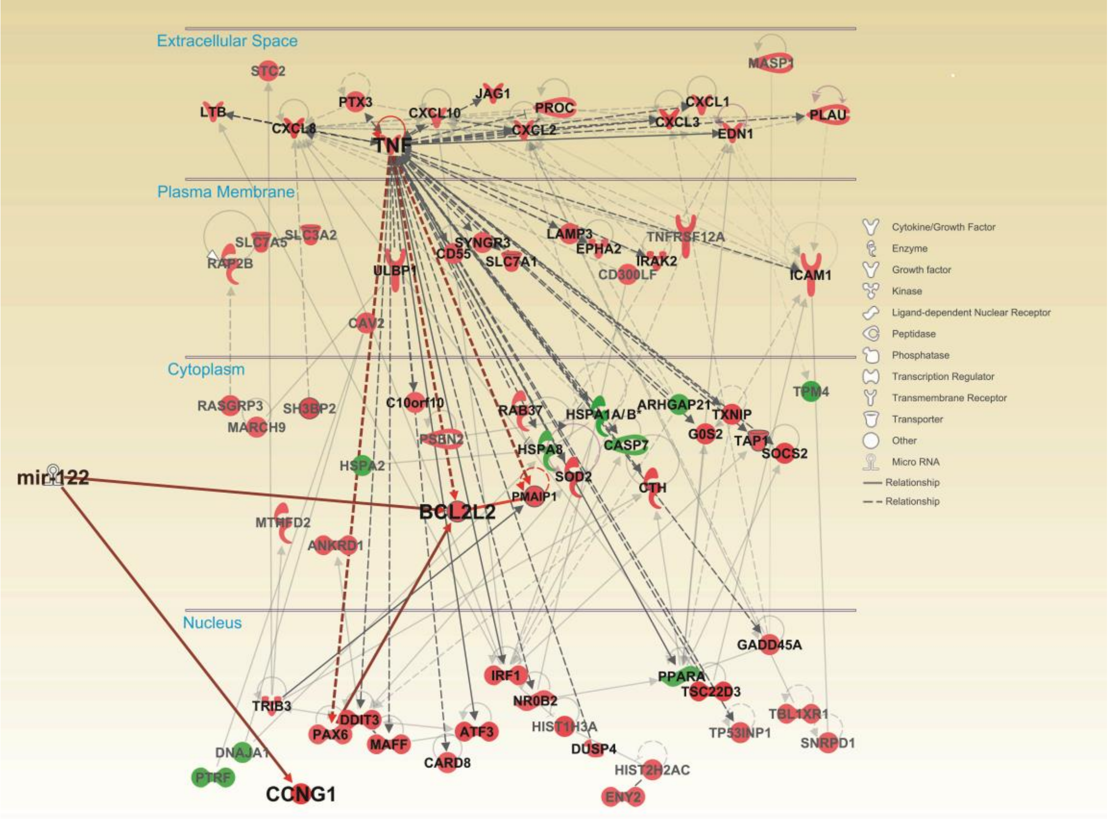

Supplementary Fig. S2　Functional analysis of differentially expressed genes identified by microarray.

According to the functional and biological connectivity, the network of differentially expressed genes (DEGs) was constructed by Ingenuity Pathway Analysis (IPA) software. This network consists of 73 DEGs with known biological interactions. Notes stand for genes, and edges for the biological relationship between them. Red and green shaded nodes represent up- and down- regulated genes, respectively; meanwhile, the solid and dashed lines represent the direct and indirect relationships, respectively. CCNG1 and BCL2L, as verified targets of mir-122, were up-regulated after mir-122 sequestration. Furthermore, TNF seems to be crucial due to having biological relationships with over 60% of all these DEGs (46/73).

Supplementary Table. S1 Conservation of HBV genome sequences within miR-122 response elements in different genotypes. The proposed interactions of miR-122 to its target sequence, which is highly conserved among 4 HBV genotypes. The seed sequence are in bold.

Genotype	MRE1691	MRE2738
	CAACGACCGACCTTGAGGCA**TACTTC**A	TAGGCATTATTTACA**TACTCT**G
**A**	———————————————————	CC—A———————————
**B**	——————————————————	CC—A———————————T
**C**	——————————————————	———————————————
**D**	——————————————————	———A—————— **C**————
